# (*E*)-3-(3,4-Di­fluoro­phen­yl)-1-(3,4-di­meth­oxy­phen­yl)prop-2-en-1-one

**DOI:** 10.1107/S1600536813013767

**Published:** 2013-05-25

**Authors:** He-Ping Zhu, Peng-Tian Yu, Zhe Wang, Sheng-Li Yang, Zhi-Guo Liu

**Affiliations:** a School of Pharmaceutical Sciences, Wenzhou Medical College, Wenzhou 325035, People’s Republic of China

## Abstract

In the title compound, C_17_H_14_F_2_O_3_, the dihedral angle between the benzene rings is 20.56 (8)° and the H atoms at the central propenone group are *trans* configured. One of the F atoms is disordered over two positions (occupancy ratio 0.57:0.43) and was refined using a split model. In the crystal, the molecules are linked into centrosymmetrical dimers and are further connected into a three-dimensional network *via* weak C—H⋯O interactions.

## Related literature

For related structures, see: Peng *et al.*(2010 ▶); Wu *et al.* (2010 ▶, 2011 ▶, 2012*b* ▶). For background to and applications of chalcones, see: Boumendjel *et al.* (2008 ▶); Kumar *et al.* (2011 ▶); Wu *et al.* (2011 ▶, 2012*a* ▶); Zhang *et al.* (2011 ▶).
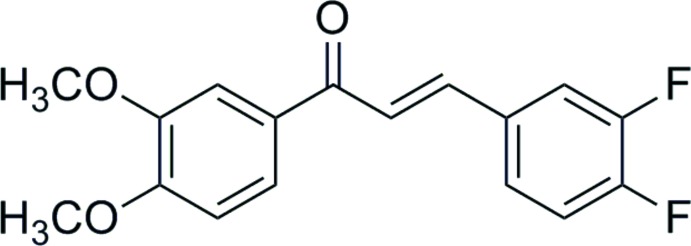


## Experimental

### 

#### Crystal data

C_17_H_14_F_2_O_3_
*M* *_r_* = 304.28
Monoclinic, 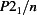

*a* = 8.7444 (9) Å
*b* = 8.4832 (9) Å
*c* = 19.829 (2) Å
β = 94.053 (2)°
*V* = 1467.2 (3) Å^3^
*Z* = 4
Mo *K*α radiation
μ = 0.11 mm^−1^
*T* = 293 K
0.21 × 0.15 × 0.11 mm

#### Data collection

Bruker SMART CCD area-detector diffractometer
Absorption correction: multi-scan (*SADABS*; Bruker, 2002 ▶) *T*_min_ = 0.977, *T*_max_ = 0.988
8592 measured reflections
2876 independent reflections
2121 reflections with *I* > 2σ(*I*)
*R*_int_ = 0.028

#### Refinement

*R*[*F* ^2^ > 2σ(*F* ^2^)] = 0.043
*wR*(*F* ^2^) = 0.129
*S* = 1.04
2876 reflections
201 parameters
H-atom parameters constrained
Δρ_max_ = 0.19 e Å^−3^
Δρ_min_ = −0.15 e Å^−3^

### 

Data collection: *SMART* (Bruker, 2002 ▶); cell refinement: *SAINT* (Bruker, 2002 ▶); data reduction: *SAINT*; program(s) used to solve structure: *SHELXS97* (Sheldrick, 2008 ▶); program(s) used to refine structure: *SHELXL97* (Sheldrick, 2008 ▶); molecular graphics: *SHELXTL* (Sheldrick, 2008 ▶); software used to prepare material for publication: *SHELXTL*.

## Supplementary Material

Crystal structure: contains datablock(s) I, global. DOI: 10.1107/S1600536813013767/nc2310sup1.cif

Structure factors: contains datablock(s) I. DOI: 10.1107/S1600536813013767/nc2310Isup2.hkl

Supplementary material file. DOI: 10.1107/S1600536813013767/nc2310Isup3.cml

Additional supplementary materials:  crystallographic information; 3D view; checkCIF report

## Figures and Tables

**Table 1:** Hydrogen-bond geometry (Å, °)

*D*—H⋯*A*	*D*—H	H⋯*A*	*D*⋯*A*	*D*—H⋯*A*
C1—H1⋯O1^i^	0.93	2.44	3.321 (2)	159
C5—H5⋯O2^ii^	0.93	2.60	3.2769 (15)	130
C5—H5⋯O3^ii^	0.93	2.49	3.3950 (15)	164

Symmetry codes: (i) 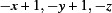; (ii) 
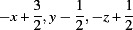
.

**Fig. 1.**
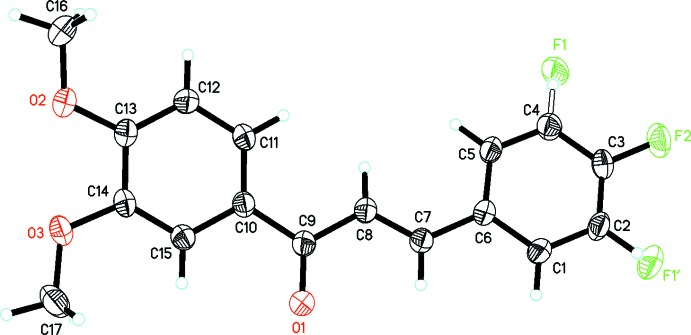
Crystal structure of the title compound with labeling and displacement ellipsoids drawn at the 30% probability level. Disordering is shown as full and open bonds.

## supplementary crystallographic information

### Comment

The title compound is a biologically active derivative of chalcone. Chalcones constitute an important group of natural products and some of them possess a wide range of biological activities including anti-inflammatory and anti-tumor (Boumendjel *et al.*, 2008; Kumar *et al.*, 2011; Zhang *et al.*, 2011). As part of our ongoing studies on chalcones (Wu *et al.*, 2012*a*,*b*; 2011), the title compound was synthesized and its crystal structure is reported here.

In the crystal structure, the dihedral angle between the mean planes of the difluorophenyl and the dimethoxyphenyl rings amount to 20.56 (8)°. The H atoms of the central propenone group are *trans* configurated. The two methoxy groups attached to C13 and C14 are almost coplanar with the benzene ring.

### Experimental

1-(3,4-difluorophenyl) ethanone (0.01 mol) and 2,3-dimethoxybenzaldehyde (0.01 mol) were dissolved in methanol (50 ml). Sodium hydroxide (5 ml, 20%) was added drop wised to the solution, which was stirred at ambient temperature. The content of the flask were poured into ice-cold water, and the resulting crude solid was collected by filtration. The compound was purified by flash column and single crystals were obtained by slow evaporation from an ethanol/ dichloromethane solution (1:2, *v*/*v*) at 293 K.

### Refinement

The hydrogen atoms were positioned with idalized geometry and refined isotropic with U_iso_(H) = 1.2 U_eq_(C) (1.5 for methyl H atoms) using a riding model. One of the fluorine atoms was disordered over two positions and was refined using a split model with sof = 0.55 and 0.45.

### Crystal data

**Table:**

C_17_H_14_F_2_O_3_	*F*(000) = 632
*M**_r_* = 304.28	*D*_x_ = 1.377 Mg m^−^^3^
Monoclinic, *P*2_1_/*n*	Mo *K*α radiation, λ = 0.71073 Å
*a* = 8.7444 (9) Å	Cell parameters from 2015 reflections
*b* = 8.4832 (9) Å	θ = 5.0–54.2°
*c* = 19.829 (2) Å	µ = 0.11 mm^−^^1^
β = 94.053 (2)°	*T* = 293 K
*V* = 1467.2 (3) Å^3^	Prismatic, colorless
*Z* = 4	0.21 × 0.15 × 0.11 mm

### Data collection

**Table:**

Bruker SMART CCD area-detector diffractometer	2876 independent reflections
Radiation source: fine-focus sealed tube	2121 reflections with *I* > 2σ(*I*)
Graphite monochromator	*R*_int_ = 0.028
phi and ω scans	θ_max_ = 26.0°, θ_min_ = 2.6°
Absorption correction: multi-scan (*SADABS*; Bruker, 2002)	*h* = −10→10
*T*_min_ = 0.977, *T*_max_ = 0.988	*k* = −10→10
8592 measured reflections	*l* = −24→20

### Refinement

**Table:**

Refinement on *F*^2^	Primary atom site location: structure-invariant direct methods
Least-squares matrix: full	Secondary atom site location: difference Fourier map
*R*[*F*^2^ > 2σ(*F*^2^)] = 0.043	Hydrogen site location: inferred from neighbouring sites
*wR*(*F*^2^) = 0.129	H-atom parameters constrained
*S* = 1.04	*w* = 1/[σ^2^(*F*_o_^2^) + (0.0703*P*)^2^ + 0.091*P*] where *P* = (*F*_o_^2^ + 2*F*_c_^2^)/3
2876 reflections	(Δ/σ)_max_ < 0.001
201 parameters	Δρ_max_ = 0.19 e Å^−^^3^
0 restraints	Δρ_min_ = −0.15 e Å^−^^3^

### Special details

**Table:**

Geometry. All e.s.d.'s (except the e.s.d. in the dihedral angle between two l.s. planes) are estimated using the full covariance matrix. The cell e.s.d.'s are taken into account individually in the estimation of e.s.d.'s in distances, angles and torsion angles; correlations between e.s.d.'s in cell parameters are only used when they are defined by crystal symmetry. An approximate (isotropic) treatment of cell e.s.d.'s is used for estimating e.s.d.'s involving l.s. planes.
Refinement. Refinement of *F*^2^ against ALL reflections. The weighted *R*-factor *wR* and goodness of fit *S* are based on *F*^2^, conventional *R*-factors *R* are based on *F*, with *F* set to zero for negative *F*^2^. The threshold expression of *F*^2^ > σ(*F*^2^) is used only for calculating *R*-factors(gt) *etc*. and is not relevant to the choice of reflections for refinement. *R*-factors based on *F*^2^ are statistically about twice as large as those based on *F*, and *R*- factors based on ALL data will be even larger.

### Fractional atomic coordinates and isotropic or equivalent isotropic displacement parameters (Å^2^)

**Table:**

	*x*	*y*	*z*	*U*_iso_*/*U*_eq_	Occ. (<1)
F1	−0.0102 (2)	−0.0304 (3)	0.14625 (12)	0.0862 (7)	0.57
F1'	−0.1205 (3)	0.2840 (4)	−0.03983 (14)	0.0844 (9)	0.43
F2	−0.21164 (11)	0.07757 (14)	0.05038 (6)	0.0743 (4)	
O1	0.67825 (13)	0.44604 (16)	0.07214 (6)	0.0643 (4)	
O2	1.05520 (12)	0.37085 (16)	0.35153 (5)	0.0569 (3)	
O3	1.13490 (13)	0.49922 (18)	0.24315 (6)	0.0711 (4)	
C1	0.12377 (18)	0.3023 (2)	0.01589 (8)	0.0512 (4)	
H1	0.1536	0.3772	−0.0148	0.061*	
C2	−0.02383 (9)	0.24437 (11)	0.01024 (4)	0.0546 (4)	
H2	−0.0955	0.2809	−0.0253	0.065*	0.57
C3	−0.06758 (9)	0.13470 (11)	0.05544 (4)	0.0520 (4)	
C4	0.03440 (9)	0.08176 (11)	0.10642 (4)	0.0557 (4)	
H4'	0.0024	0.0047	0.1379	0.067*	0.43
C5	0.18143 (9)	0.13853 (11)	0.11244 (4)	0.0520 (4)	
H5	0.2498	0.1023	0.1471	0.062*	
C6	0.22850 (16)	0.24987 (19)	0.06709 (8)	0.0431 (4)	
C7	0.38359 (17)	0.3175 (2)	0.07232 (8)	0.0457 (4)	
H7	0.4064	0.3889	0.0389	0.055*	
C8	0.49391 (17)	0.2877 (2)	0.11943 (8)	0.0505 (4)	
H8	0.4769	0.2139	0.1528	0.061*	
C9	0.64366 (17)	0.3684 (2)	0.12068 (8)	0.0474 (4)	
C10	0.74973 (16)	0.35850 (19)	0.18235 (8)	0.0441 (4)	
C11	0.71003 (19)	0.2904 (2)	0.24136 (9)	0.0588 (5)	
H11	0.6145	0.2429	0.2426	0.071*	
C12	0.80881 (19)	0.2909 (2)	0.29899 (9)	0.0587 (5)	
H12	0.7792	0.2439	0.3384	0.070*	
C13	0.95050 (17)	0.36056 (19)	0.29820 (8)	0.0459 (4)	
C14	0.99356 (16)	0.42987 (19)	0.23785 (8)	0.0458 (4)	
C15	0.89489 (17)	0.42856 (18)	0.18126 (8)	0.0449 (4)	
H15	0.9242	0.4746	0.1416	0.054*	
C16	1.0133 (2)	0.3151 (3)	0.41527 (9)	0.0700 (6)	
H16A	0.9196	0.3648	0.4263	0.105*	
H16B	1.0931	0.3399	0.4494	0.105*	
H16C	0.9990	0.2030	0.4132	0.105*	
C17	1.1804 (2)	0.5882 (3)	0.18825 (10)	0.0762 (6)	
H17A	1.1838	0.5214	0.1493	0.114*	
H17B	1.2802	0.6320	0.1993	0.114*	
H17C	1.1083	0.6719	0.1786	0.114*	

### Atomic displacement parameters (Å^2^)

**Table:**

	*U*^11^	*U*^22^	*U*^33^	*U*^12^	*U*^13^	*U*^23^
F1	0.0593 (11)	0.0901 (16)	0.1083 (16)	−0.0182 (10)	0.0008 (10)	0.0475 (13)
F1'	0.0602 (14)	0.111 (2)	0.0770 (17)	−0.0076 (14)	−0.0306 (13)	0.0194 (16)
F2	0.0443 (5)	0.0795 (8)	0.0971 (9)	−0.0160 (5)	−0.0097 (5)	0.0008 (6)
O1	0.0498 (7)	0.0841 (10)	0.0580 (7)	−0.0110 (6)	−0.0045 (6)	0.0218 (7)
O2	0.0488 (6)	0.0697 (8)	0.0502 (7)	−0.0076 (6)	−0.0095 (5)	−0.0012 (6)
O3	0.0497 (7)	0.1028 (11)	0.0602 (8)	−0.0332 (7)	−0.0014 (6)	0.0012 (7)
C1	0.0492 (9)	0.0576 (11)	0.0454 (9)	−0.0017 (8)	−0.0057 (7)	0.0055 (8)
C2	0.0453 (9)	0.0650 (12)	0.0510 (10)	0.0007 (8)	−0.0140 (8)	0.0014 (8)
C3	0.0369 (8)	0.0538 (10)	0.0644 (10)	−0.0046 (7)	−0.0044 (7)	−0.0089 (8)
C4	0.0490 (9)	0.0506 (11)	0.0668 (11)	−0.0037 (8)	−0.0002 (8)	0.0092 (8)
C5	0.0431 (9)	0.0564 (11)	0.0550 (10)	0.0024 (8)	−0.0076 (7)	0.0088 (8)
C6	0.0403 (8)	0.0445 (9)	0.0436 (8)	0.0022 (7)	−0.0032 (6)	−0.0026 (7)
C7	0.0432 (8)	0.0498 (10)	0.0438 (9)	−0.0001 (7)	0.0007 (7)	−0.0001 (7)
C8	0.0420 (8)	0.0550 (10)	0.0534 (10)	−0.0056 (7)	−0.0052 (7)	0.0080 (8)
C9	0.0404 (8)	0.0498 (10)	0.0514 (9)	−0.0002 (7)	−0.0006 (7)	0.0047 (8)
C10	0.0381 (8)	0.0437 (9)	0.0498 (9)	−0.0031 (7)	−0.0014 (7)	0.0007 (7)
C11	0.0442 (9)	0.0694 (12)	0.0616 (11)	−0.0195 (8)	−0.0055 (8)	0.0140 (9)
C12	0.0544 (10)	0.0688 (12)	0.0515 (10)	−0.0157 (9)	−0.0053 (8)	0.0157 (8)
C13	0.0422 (8)	0.0449 (9)	0.0496 (9)	−0.0010 (7)	−0.0050 (7)	−0.0035 (7)
C14	0.0364 (8)	0.0472 (10)	0.0533 (9)	−0.0065 (7)	0.0005 (7)	−0.0070 (7)
C15	0.0430 (8)	0.0454 (9)	0.0465 (9)	−0.0033 (7)	0.0041 (7)	−0.0017 (7)
C16	0.0654 (11)	0.0901 (15)	0.0523 (11)	−0.0033 (10)	−0.0120 (9)	0.0085 (10)
C17	0.0639 (12)	0.0933 (16)	0.0735 (13)	−0.0337 (11)	0.0201 (10)	−0.0104 (11)

### Geometric parameters (Å, º)

**Table:**

F1—C4	1.313 (2)	C7—H7	0.9300
F1'—C2	1.302 (2)	C8—C9	1.476 (2)
F2—C3	1.3468 (12)	C8—H8	0.9300
O1—C9	1.2220 (19)	C9—C10	1.484 (2)
O2—C13	1.3516 (17)	C10—C11	1.371 (2)
O2—C16	1.421 (2)	C10—C15	1.403 (2)
O3—C14	1.3662 (18)	C11—C12	1.383 (2)
O3—C17	1.405 (2)	C11—H11	0.9300
C1—C2	1.3784 (18)	C12—C13	1.374 (2)
C1—C6	1.391 (2)	C12—H12	0.9300
C1—H1	0.9300	C13—C14	1.408 (2)
C2—C3	1.3650	C14—C15	1.366 (2)
C2—H2	0.9600	C15—H15	0.9300
C3—C4	1.3753	C16—H16A	0.9600
C4—C5	1.3703	C16—H16B	0.9600
C4—H4'	0.9600	C16—H16C	0.9600
C5—C6	1.3866 (18)	C17—H17A	0.9600
C5—H5	0.9300	C17—H17B	0.9600
C6—C7	1.469 (2)	C17—H17C	0.9600
C7—C8	1.319 (2)		
			
C13—O2—C16	118.12 (13)	C8—C9—C10	119.32 (14)
C14—O3—C17	118.42 (14)	C11—C10—C15	118.39 (14)
C2—C1—C6	120.61 (15)	C11—C10—C9	123.07 (14)
C2—C1—H1	119.7	C15—C10—C9	118.47 (14)
C6—C1—H1	119.7	C10—C11—C12	121.53 (15)
F1'—C2—C3	118.67 (13)	C10—C11—H11	119.2
F1'—C2—C1	121.57 (16)	C12—C11—H11	119.2
C3—C2—C1	119.62 (8)	C13—C12—C11	120.20 (16)
C3—C2—H2	120.2	C13—C12—H12	119.9
C1—C2—H2	120.2	C11—C12—H12	119.9
F2—C3—C2	120.03 (6)	O2—C13—C12	125.48 (15)
F2—C3—C4	119.45 (6)	O2—C13—C14	115.52 (13)
C2—C3—C4	120.5	C12—C13—C14	118.99 (14)
F1—C4—C5	121.27 (9)	O3—C14—C15	125.60 (15)
F1—C4—C3	118.29 (9)	O3—C14—C13	114.15 (13)
C5—C4—C3	120.3	C15—C14—C13	120.22 (13)
C5—C4—H4'	119.8	C14—C15—C10	120.68 (15)
C3—C4—H4'	119.8	C14—C15—H15	119.7
C4—C5—C6	120.17 (6)	C10—C15—H15	119.7
C4—C5—H5	119.9	O2—C16—H16A	109.5
C6—C5—H5	119.9	O2—C16—H16B	109.5
C5—C6—C1	118.75 (13)	H16A—C16—H16B	109.5
C5—C6—C7	122.34 (12)	O2—C16—H16C	109.5
C1—C6—C7	118.89 (14)	H16A—C16—H16C	109.5
C8—C7—C6	126.89 (15)	H16B—C16—H16C	109.5
C8—C7—H7	116.6	O3—C17—H17A	109.5
C6—C7—H7	116.6	O3—C17—H17B	109.5
C7—C8—C9	121.78 (15)	H17A—C17—H17B	109.5
C7—C8—H8	119.1	O3—C17—H17C	109.5
C9—C8—H8	119.1	H17A—C17—H17C	109.5
O1—C9—C8	120.35 (14)	H17B—C17—H17C	109.5
O1—C9—C10	120.29 (14)		
			
C6—C1—C2—F1'	−175.4 (2)	O1—C9—C10—C11	171.47 (18)
C6—C1—C2—C3	0.14 (19)	C8—C9—C10—C11	−6.5 (3)
F1'—C2—C3—F2	−4.45 (17)	O1—C9—C10—C15	−5.4 (2)
C1—C2—C3—F2	179.90 (13)	C8—C9—C10—C15	176.53 (15)
F1'—C2—C3—C4	175.63 (17)	C15—C10—C11—C12	0.5 (3)
C1—C2—C3—C4	−0.03 (9)	C9—C10—C11—C12	−176.42 (17)
F2—C3—C4—F1	3.98 (15)	C10—C11—C12—C13	0.0 (3)
C2—C3—C4—F1	−176.10 (14)	C16—O2—C13—C12	−4.5 (3)
F2—C3—C4—C5	−179.96 (7)	C16—O2—C13—C14	174.74 (16)
C2—C3—C4—C5	0.0	C11—C12—C13—O2	178.70 (16)
F1—C4—C5—C6	175.92 (18)	C11—C12—C13—C14	−0.5 (3)
C3—C4—C5—C6	−0.02 (8)	C17—O3—C14—C15	5.4 (3)
C4—C5—C6—C1	0.13 (17)	C17—O3—C14—C13	−172.30 (16)
C4—C5—C6—C7	178.41 (10)	O2—C13—C14—O3	−0.9 (2)
C2—C1—C6—C5	−0.2 (2)	C12—C13—C14—O3	178.39 (16)
C2—C1—C6—C7	−178.53 (13)	O2—C13—C14—C15	−178.80 (14)
C5—C6—C7—C8	−2.0 (3)	C12—C13—C14—C15	0.5 (2)
C1—C6—C7—C8	176.24 (16)	O3—C14—C15—C10	−177.60 (16)
C6—C7—C8—C9	−177.52 (15)	C13—C14—C15—C10	0.0 (2)
C7—C8—C9—O1	−11.8 (3)	C11—C10—C15—C14	−0.5 (2)
C7—C8—C9—C10	166.18 (16)	C9—C10—C15—C14	176.55 (15)

### Hydrogen-bond geometry (Å, º)

**Table:**

*D*—H···*A*	*D*—H	H···*A*	*D*···*A*	*D*—H···*A*
C1—H1···O1^i^	0.93	2.44	3.321 (2)	159
C5—H5···O2^ii^	0.93	2.60	3.2769 (15)	130
C5—H5···O3^ii^	0.93	2.49	3.3950 (15)	164

Symmetry codes: (i) −*x*+1, −*y*+1, −*z*; (ii) −*x*+3/2, *y*−1/2, −*z*+1/2.

## References

[bb1] Boumendjel, A., Boccard, J., Carrupt, P. A., Nicolle, E., Blanc, M., Geze, A., Choisnard, L., Wouessidjewe, D., Matera, E. L. & Dumontet, C. (2008). *J. Med. Chem.* **51**, 2307–2310. 10.1021/jm0708331 18293907

[bb2] Bruker (2002). *SMART*, *SAINT* and *SADABS* Bruker AXS Inc., Madison, Wisconsin, USA.

[bb3] Kumar, V., Kumar, S., Hassan, M., Wu, H., Thimmulappa, R. K., Kumar, A., Sharma, S. K., Parmar, V. S., Biswal, S. & Malhotra, S. V. (2011). *J. Med. Chem.* **54**, 4147–4159. 10.1021/jm2002348 PMC3212436 21539383

[bb4] Peng, J., Xu, H., Li, Z., Zhang, Y. & Wu, J. (2010). *Acta Cryst.* E**66**, o1156–o1157. 10.1107/S1600536810014169 PMC2979023 21579201

[bb5] Sheldrick, G. M. (2008). *Acta Cryst.* A**64**, 112–122. 10.1107/S0108767307043930 18156677

[bb6] Wu, X., Cai, X., Zheng, X., Zhang, Z. & Ye, X. (2010). *Acta Cryst.* E**66**, o3015. 10.1107/S1600536810043606 PMC3009186 21589174

[bb7] Wu, J. Z., Jiang, X., Zhao, C. G., Li, X. K. & Yang, S. L. (2012*b*). *Z. Kristallogr. New Cryst. Struct.* **227**, 215–216.

[bb8] Wu, J. Z., Li, J. L., Cai, Y. P., Pan, Y., Ye, F. Q., Zhang, Y. L., Zhao, Y. J., Yang, S. L., Li, X. K. & Liang, G. (2011). *J. Med. Chem* **54**, 8110–8123. 10.1021/jm200946h 21988173

[bb9] Wu, J. Z., Wang, C., Cai, Y. P., Peng, J., Liang, D. L., Zhao, Y. J., Yang, S. L., Li, X. K., Wu, X. P. & Liang, G. (2012*a*). *Med. Chem. Res.* **21**, 444–452.

[bb10] Zhang, H. J., Qian, Y., Zhu, D. D., Yang, X. G. & Zhu, H. L. (2011). *Eur. J. Med. Chem.* **46**, 4702–4708. 10.1016/j.ejmech.2011.07.016 21816517

